# A Simplified Model of the Knee for Coronal Plane Alignment of the Knee Simulation

**DOI:** 10.3390/bioengineering13080946

**Published:** 2026-08-21

**Authors:** César Rodríguez Pereira, Javier Gracia Rodríguez, Fernando Sánchez Lasheras, Francisco Javier Iglesias Rodríguez, Antonio Murcia Asensio

**Affiliations:** 1 Instituto de Ciencias y Tecnologías Espaciales de Asturias, University of Oviedo, 33004 Oviedo, Spaingraciajavier@uniovi.es (J.G.R.); fjiglesias@uniovi.es (F.J.I.R.); 2 Department of Manufacturing and Construction Engineering, University of Oviedo, 33203 Gijón, Spain; 3 Department of Mathematics, University of Oviedo, 33007 Oviedo, Spain; 4 Hospital General Universitario Reina Sofía, Avenida Intendente Jorge Palacios 1, 30003 Murcia, Spain; amurciasensio@gmail.com

**Keywords:** adjacent effect, knee joint, FEM, CPAK

## Abstract

A clinical observation of a previously asymptomatic native knee developing a relevant coronal alignment change after total hip arthroplasty (THA) motivated this research. This raised the question of whether the native knee morphology could influence the response to biomechanical changes elsewhere in the lower limb. First, we developed a simplified finite element model incorporating the Coronal Plane Alignment of the Knee (CPAK) classification. We developed a simplified model of the knee, where we represented the lateral distal femoral angle (LDFA) by physically rotating the femur, and to avoid remodeling the tibia coronal plane for each simulation, we represented the medial proximal tibial angle (MPTA) by adjusting the neutral angle of the joint (the angle at which the joint generates no reaction force). This allowed for quick parametric iteration over the CPAK input space, showing which type of knees were more vulnerable to extreme valgus (CPAK III) and varus (CPAK VII) due to their morphology. The simplicity of this model will also enable users with less technical expertise to quickly obtain insights into specific knee morphologies, without requiring deep knowledge about finite element modeling. This existing model lays the foundation for later work, with a deeper look into how changes to hip morphology build on the pre-existing tendencies to varus and valgus.

## 1. Introduction

Surgical interventions aimed at correcting deformities, restoring joint function, or stabilizing skeletal segments (such as arthroplasties, angular corrections, or arthrodesis) have proven effective in easing pain and improving function in patients with advanced joint pathologies. However, these mechanical and structural modifications often significantly alter neighboring joints, affecting their alignment, biomechanics, and clinical functionality. This is known as the adjacent effect (AE) and is widely documented in the existing literature, highlighting how changes in one joint can have profound repercussions for the biomechanical integrity of nearby structures.

The adjacent effect, analogous to the butterfly effect described by Edward Lorenz [1], emphasizes how small modifications in one part of a system can produce large-scale changes in distant areas. For instance, Cheal et al. (1992) demonstrated that alterations in proximal femoral moments following total hip arthroplasty (THA) could disrupt knee joint stress distributions, triggering bone remodeling phenomena that compromise joint stability [2].

Similarly, angular corrections during total knee arthroplasty (TKA) have been shown to influence adjacent joints. Shichman et al. (2022) reported that significant angular corrections during TKA could induce coronal alignment changes in the ankle, leading to increased pain incidence and accelerated osteoarthritis progression in the distal joint [3].

Advancements in computational modeling, particularly finite element analysis (FEA), have provided critical insights into these biomechanical interactions. Studies have shown that changes in proximal femoral geometry, such as the neck-shaft angle and anteversion angle, significantly impact the forces transmitted through total hip arthroplasty (THA) prostheses and indirectly affect knee biomechanics, potentially accelerating the progression of osteoarthritis [4,5]. Robbins et al. (2024) analyzed how the combination of THA and lumbar fusion impacts pelvic and lumbar mobility, correlating these changes with knee joint mechanics during basic functional tasks [6].

The Coronal Plane Alignment of the Knee (CPAK) classification helps us to better understand how alignment changes biomechanically impact knee functionality. CPAK introduces a systematic approach to characterizing knee alignment phenotypes, incorporating parameters such as the arithmetic hip–knee–ankle angle (aHKA) and joint line obliquity (JLO). This classification enables surgeons and researchers to identify the knee phenotypes most susceptible to biomechanical imbalances [7].

FEA provides a computational framework for investigating complex biomechanical systems, including the lower limb, by estimating stress distributions, load transfer, and mechanical responses under defined loading and boundary conditions. The credibility of these predictions depends on transparent model reporting and appropriate verification and validation procedures [8].

Preoperative planning for total hip arthroplasty generally focuses on restoring hip anatomy, whereas the coronal alignment and anatomical characteristics of the ipsilateral native knee may not always be fully characterized. THA may alter lower-limb biomechanics through changes in femoral offset, limb length, hip center position, femoral lever ratios, implant design, and muscle or soft-tissue function related to the surgical approach [9].

From a clinical perspective, this problem is particularly relevant because the ipsilateral knee may be completely asymptomatic at the time of THA and its native coronal phenotype may therefore not be specifically assessed. Nevertheless, a clinically silent knee may have an alignment pattern that makes it less tolerant of changes in lower-limb mechanics following hip reconstruction. This clinical observation raises the possibility that the response to THA may depend not only on how the hip is reconstructed, but also on the pre-existing morphology of the ipsilateral knee.

The clinical question is therefore not simply whether THA can alter ipsilateral knee alignment, but which native knees are most susceptible to these changes and which components of hip reconstruction have the greatest influence. It remains unknown whether specific CPAK phenotypes are particularly vulnerable to changes in femoral offset, limb length, hip center position, or other reconstruction variables. Identifying these relationships could improve the preoperative assessment of the ipsilateral knee, support individualized patient counseling and informed consent, and contribute to patient-specific THA planning. If specific phenotypes are eventually shown to be at greater risk, greater attention could be directed toward the reconstruction parameters to which their native knee phenotype is most sensitive.

Addressing these clinical questions first requires a biomechanical model capable of reproducing different CPAK-defined knee phenotypes under controlled conditions. Therefore, the purpose of the present study was to develop a finite element model of the lower limb incorporating the CPAK classification and to characterize the coronal response of different native knee phenotypes. This study represents the first step of a broader research program aimed at evaluating how individual THA reconstruction parameters affect ipsilateral knee alignment and whether specific CPAK phenotypes are particularly vulnerable to these changes.

## 2. Materials and Methods

### 2.1. Modeling the Knee and CPAK Conceptualization

**Figure 1:**
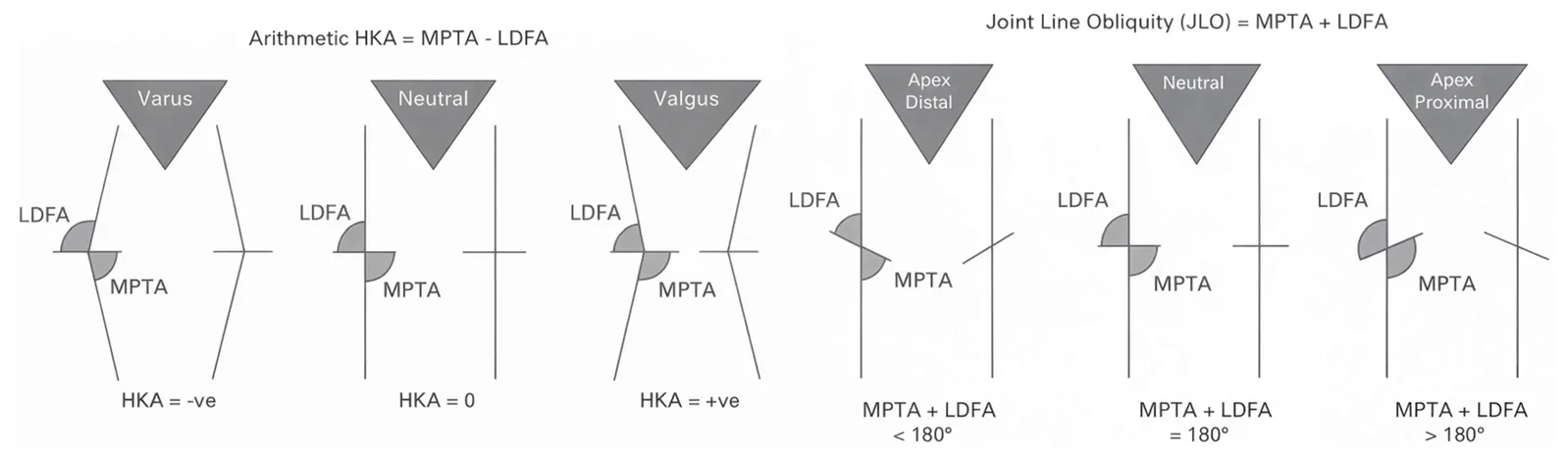
Relationship between LDFA and MPTA and the different CPAK parameters (MacDessi et al.) [7].

To study the effects of the different CPAK phenotypes, we developed a simplified model of the knee, with a heavy focus on easy parametrization of the knee morphology. The CPAK classification stems from two parameters, hip–knee–ankle angle (aHKA) and joint line obliquity (JLO). These, in turn, come from a direct relationship between two angles—the lateral distal femoral angle (LDFA) and the medial proximal tibial angle (MPTA)—represented in Figure 1.

Authors such as Lerner et al. (2015) have represented the knee joint as a set of revolute joints, one acting in the sagittal plane, allowing for knee flexion, and two more acting in the frontal plane, used to resolve the frontal plane movements across the tibiofemoral joint [10]. Given that the focus of our model was to resolve the movements along this frontal plane (varus and valgus displacements), detailed reaction forces in the bones were less important, allowing us to simplify that set of revolute joints down to a single general joint.

A general joint is a 2-node element without fixed degrees of freedom by default, which allows full customization of the stiffness matrix. This type of element enables full representation of the overall knee joint mechanics, in terms of the compressive strength and rotational stiffness along the three axes, as well as cross-plane stiffnesses. However, as we focused on lateral motions, in this first work, we set large stiffnesses for every degree of freedom except the varus–valgus rotation, simulating the resistance of the medial and lateral compartments to rotation in the frontal plane. Future work may include a more in-depth look at the interactions between the different movements of the knee.

With this general framework for the knee, we aimed to parametrize CPAK in such a way that required little geometry modification. In a perfect one-to-one representation, both the LDFA and MPTA depend on the bone morphology around the knee and overall hip–knee–ankle alignment. To minimize 3D remodeling of the femur and tibial heads, we arrived at a simplified representation: the LDFA was represented through rotation of the femur above the knee, while the MPTA was represented as a change in the stable, 0-torque reaction angle of the general joint. In this way, both CPAK parameters, aHKA and JLO, were fully represented half through a simple change in geometry and half through a parameter change in the behavior of the joint, removing the need for 3D remodeling, thus improving our ability to explore the input space. The ability to run different cases by only modifying a numerical parameter also enables users with less technical expertise to independently run cases for a specific knee morphology, without the need for 3D CAD or FEM meshing knowledge.

In order to change the “neutral angle” of the joint (the angle at which it exerts no reaction), allowing the simulation of obliquity without 3D modeling, the frontal rotational stiffness of the general joint was input as tabular data, something usually reserved for nonlinear behavior. While the data used was fully linear (aside from the shift in 0-torque angle), this methodology would allow for nonlinear stiffness, which could be useful for representing certain scenarios (e.g., a knee with vastly different stiffnesses between varus and valgus motions).

Finally, there is the key aspect of which rotational stiffness to apply to the joint. Based on in vivo tests, a value of 1 N·m/deg was applied to all models [11,12]. This number is substantially less than the average value measured in men, but is only slightly below the average for women, and is within the standard deviation for the population based on both studies. We made the decision to use values toward the least stiff values within the normal range, with the intention of studying a subset of the population that is more likely to develop issues.

### 2.2. FEA Model Development

While the knee joint is the core of the model, in this section, we aim to cover the rest of the FEA model. We generated a simplified geometry of the tibia, fibula, and femur. Both the tibia and fibula were assigned a “no displacement” boundary condition on their distal end, to simulate a planted foot [13]. Femoral displacement was restricted only at its head, being prevented from moving right or left (simulating the insertion into the acetabulum). In this same place, we applied a vertical load, regardless of the aHKA angle.

**Figure 2:**
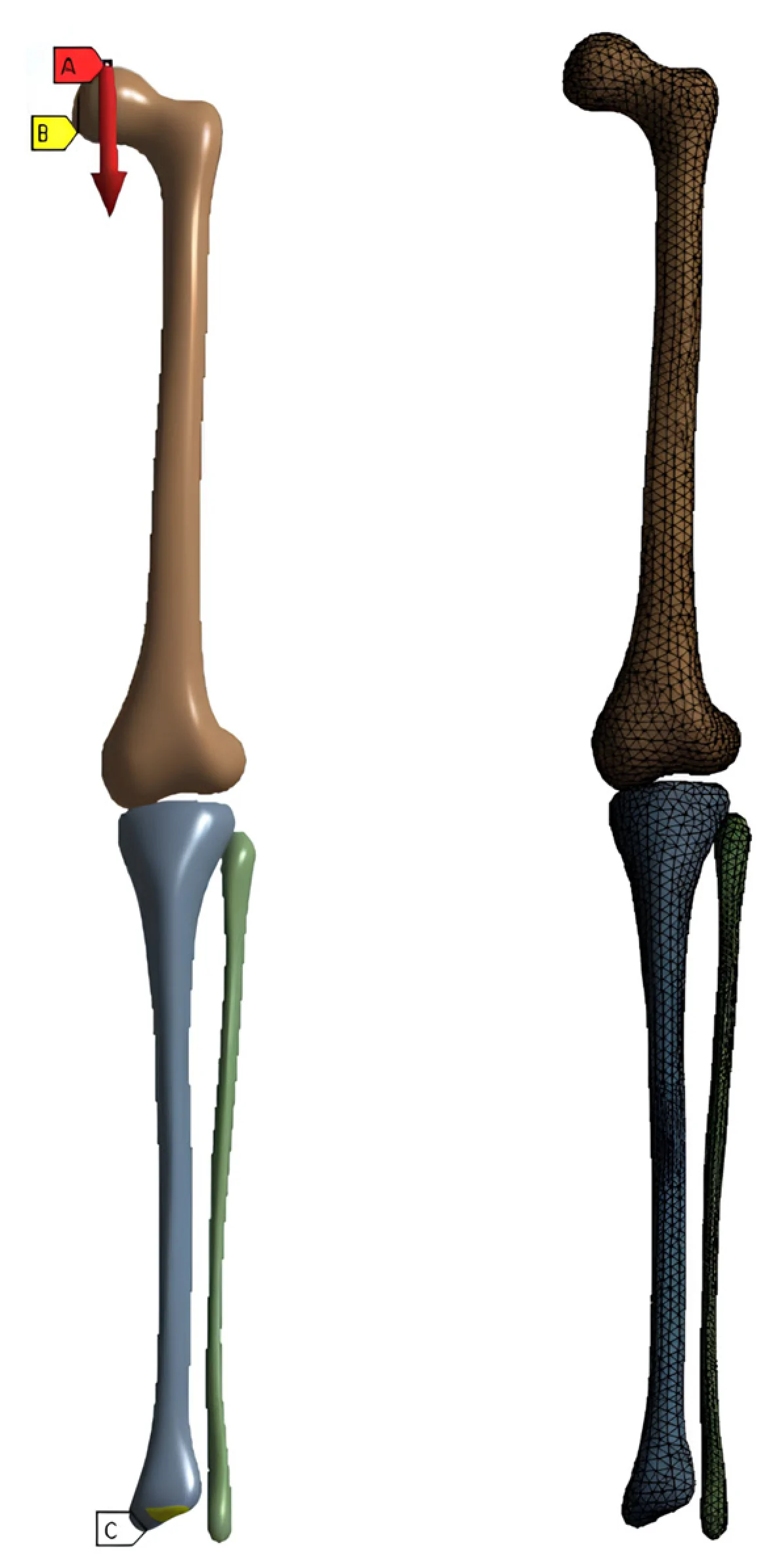
General geometry and applied boundary conditions (**left**) and model mesh (**right**). [A] Vertical load simulating patient weight. [B] Lateral movement restriction. [C] No displacement boundary.

An initial mesh was created through the default settings from Ansys Mechanical, which was later refined until the nodal stress and deformations did not significantly change upon further refinement. The resulting mesh can be seen in Figure 2.

All bones were modeled with quadratic solid tetrahedral elements. A linear isotropic elastic material was used, representing cortical bone, with a Young’s modulus of 17 GPa, and a Poisson ratio of 0.3. These numbers are in line with those used by multiple authors [13,14,15]. Large deflection effects were taken into account.

### 2.3. Model Validation

Given that the aim of this model was not to replicate an in vivo subject, but rather to represent trends and sensitivities, no physical validation was performed. This is in line with the usual approach for this type of model [16].

**Figure 3:**
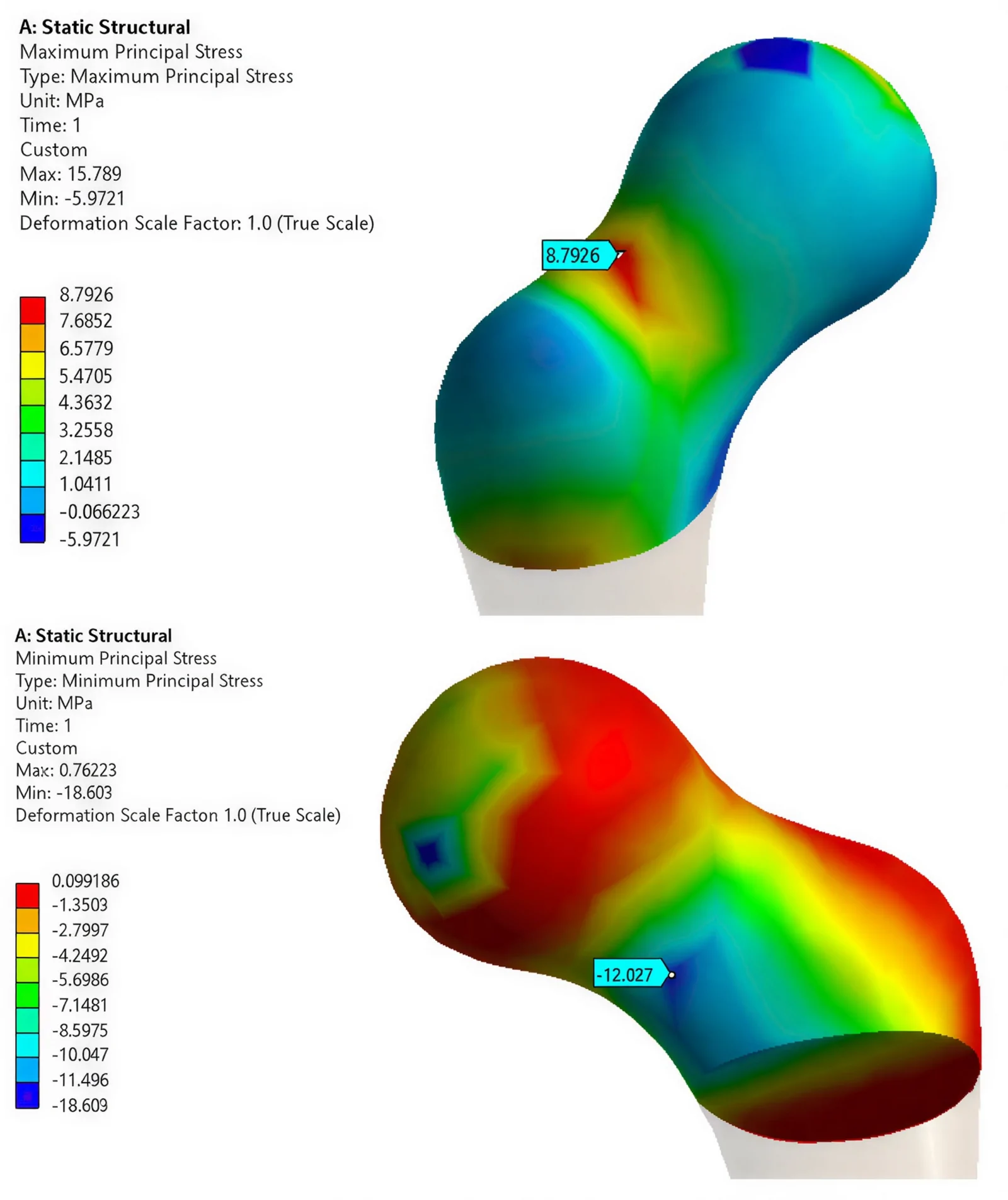
Maximum principal and minimum principal stresses from an 8° aHKA varus knee model. Despite different geometry and meshing, similar stresses appear in [17].

However, to ensure the validity of the model, stresses were compared to those of existing models [17,18]. Although we may expect to find differences due to variations in geometry, mesh density, and conceptual model between the different analyses, sufficient similarities in terms of peak stress and stress distribution arose, which suggested that the model accurately represented the processes involved. The maximum and minimum principal stress distributions obtained for the 8° aHKA varus knee model are shown in Figure 3, illustrating the similarities in stress magnitude and distribution with previously published finite elements models [17,18].

## 3. Results

We performed a uniform parametric sweep of the CPAK input space. A total of 56 models were run in total, covering arithmetic hip–knee–ankle angle values of −8° (varus knee) to +6° (valgus knee), in increments of 2 degrees. Along the other CPAK axis, joint line obliquity values of 171° (apex distal) to 189° (apex proximal) were explored.

**Figure 4:**
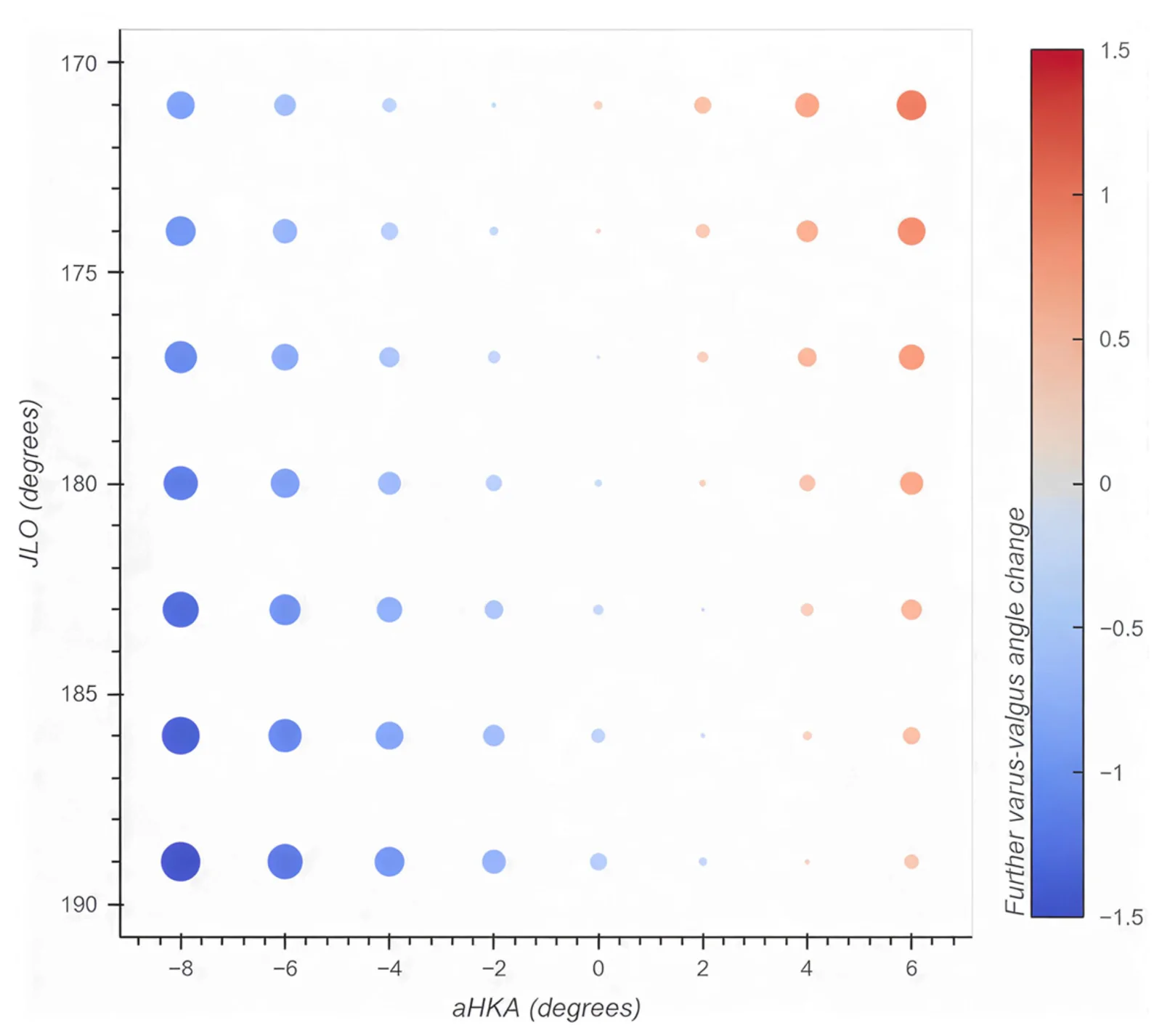
Results of the 56 models overlaid with their relative positions in CPAK. Position represents input aHKA and JLO. Dot size represents absolute rotation magnitude, while the color bar represents rotation, with red meaning further varus, and blue meaning further valgus.

The metric that we focused on in this study was the additional knee rotation: how much the knee further rotated from the starting angle, to analyze whether a given combination of aHKA and JLO caused further valgus/varus knee or whether it neutralized it. The distribution of the additional knee rotation across the complete CPAK input space is summarized in Figure 4, which shows both the magnitude and direction of the rotational response for ach combination of of aHKA and JLO.

**Figure 5:**
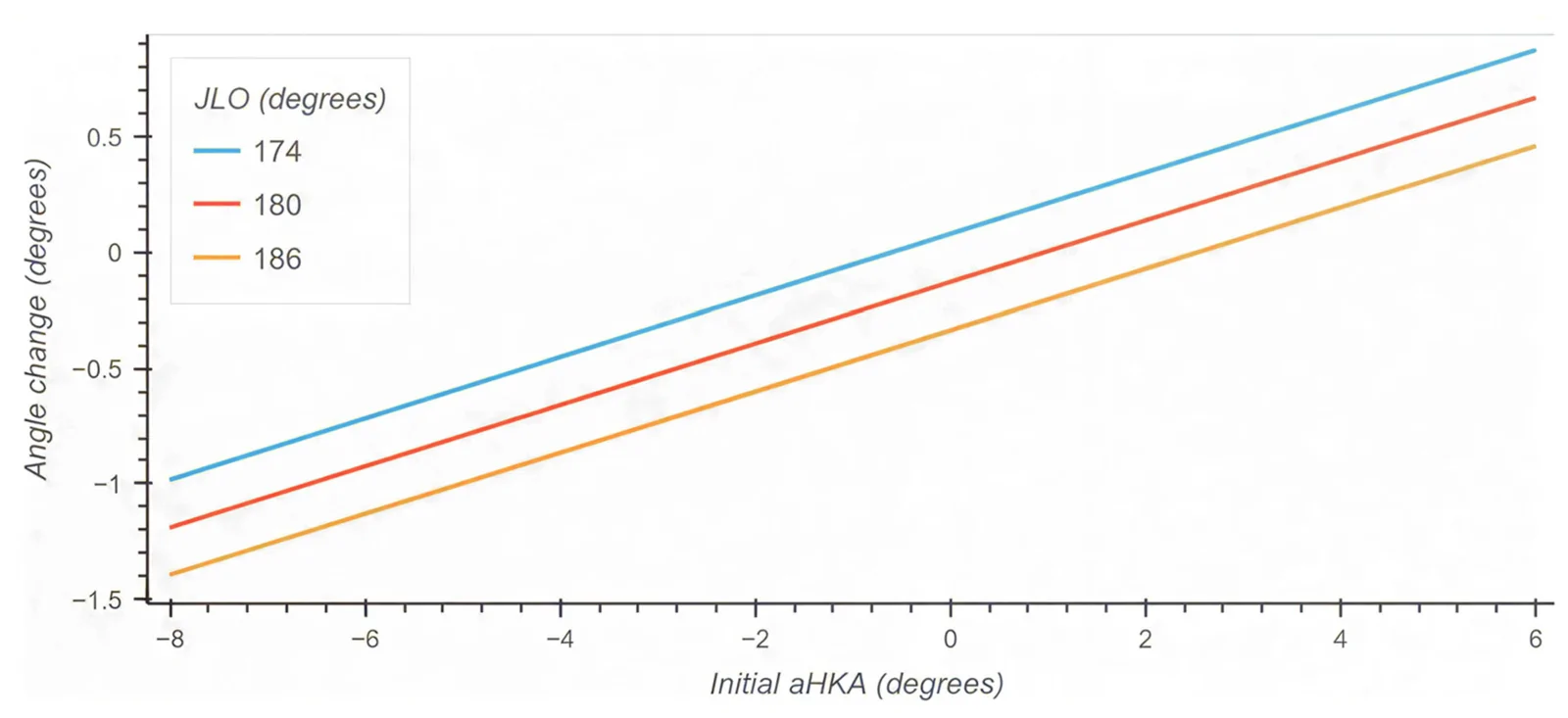
Linear behavior along any JLO or aHKA, as may be expected due to using linear properties, even if the solver configuration was set to take into account nonlinearities.

If we take a slice along any of the axes, one can see mostly linear behavior, with further initial angles or obliquities causing proportionally greater rotations, as shown in Figure 5.

**Figure 6:**
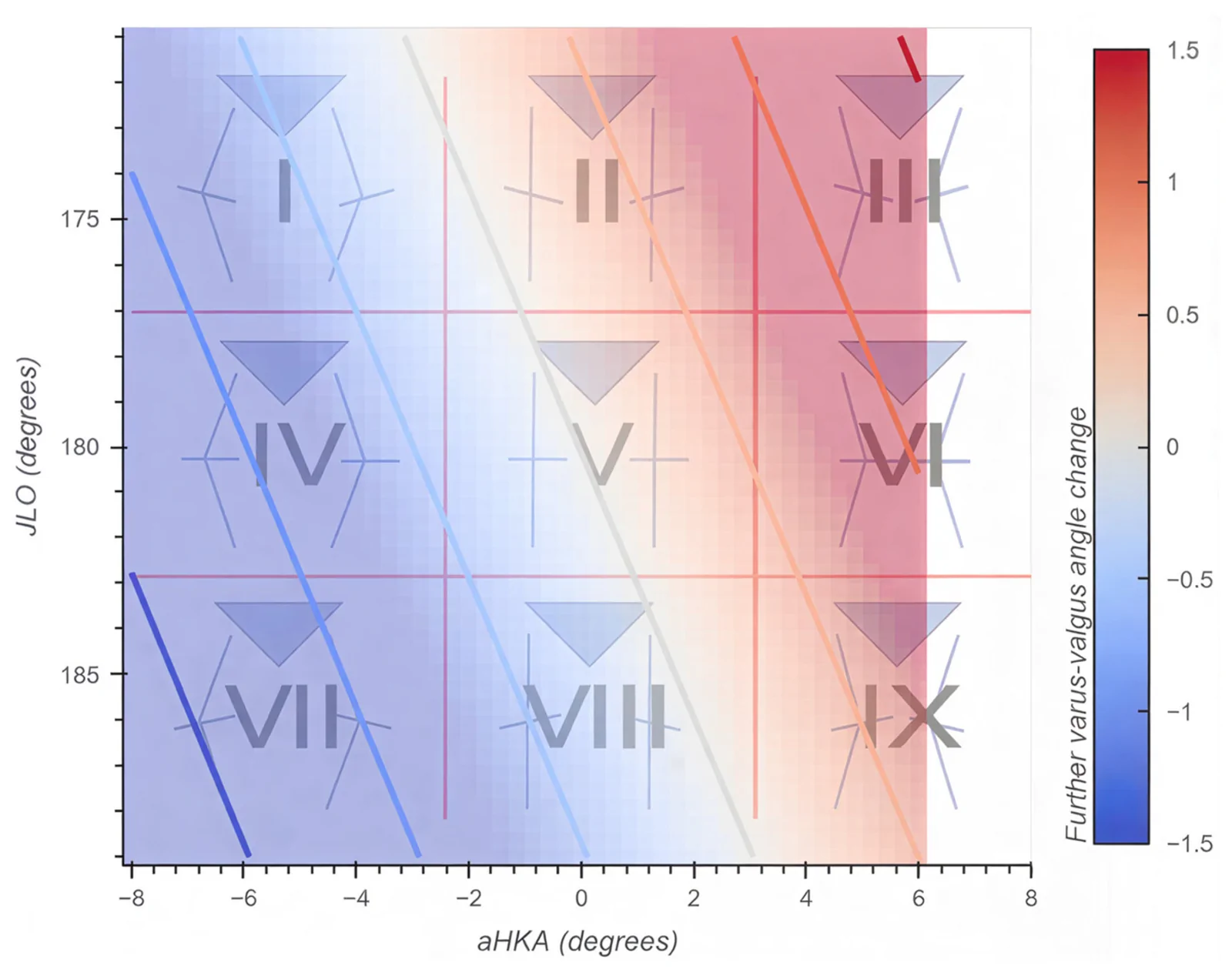
Polynomial best fit surface for varus–valgus rotation given for each initial point in the CPAK classification. Overlaid, horizontal displacement contours at the knee joint (mm), as an alternative representation of the varus and valgus tendency of each alignment.

With these results, a polynomial best fit surface of the data (Figure 6) was calculated, giving an approximate continuous response for the CPAK classification (with every other parameter, such as load and femur/tibia length, being the same).

## 4. Discussion

Current evidence documents adjacent effects following lower-limb interventions, yet how native knee alignment dictates joint tolerance to altered mechanics remains uncharacterized. Addressing this gap is essential for anticipating adverse postoperative joint changes and refining patient-specific planning. To establish this foundation, the primary objective of this work was to implement a simplified CPAK-based finite element model to quantify the intrinsic coronal behavior and stability of different native knee phenotypes.

The main clinically relevant finding of this study was that CPAK-defined native knee phenotypes did not respond in the same way under identical mechanical conditions. Some phenotypes showed a greater tendency to increase their initial varus or valgus alignment, whereas others showed smaller changes. Although THA is not yet simulated in the present model, these differences may help to explain the clinical observation that motivated this work: some previously asymptomatic knees remain asymptomatic after hip reconstruction, whereas others may become symptomatic when lower-limb mechanics are modified. Clinical evidence also suggests that THA may influence ipsilateral knee alignment [9]. However, current clinical studies do not identify which native knee phenotypes are most susceptible or which specific reconstruction parameters are responsible for these changes. The present findings begin to address the first of these questions by suggesting that native coronal knee morphology itself may influence the biomechanical response.

The results show displacements and rotations that are small compared to those expected in cases of pathological knee valgus or varus. This was anticipated, as we only studied the frontal plane contribution to these movements. Actual knee valgus and varus is a process which also involves knee flexion and usually some degree of hip internal/external rotation. Thus, these numbers should not be considered to be the total effect of coronal plane alignment on these movements but rather, a single factor that contributes to the overall valgus or varus through initial instability.

The response surface shown in Figure 6 offers relevant insight into knee mechanics. As anticipated, a knee that is neither oblique nor varus or valgus in origin tended to stay neutral, because there was no load eccentricity for which the knee must compensate. However, it is notable that there was a marked interaction between aHKA and JLO, with certain obliquities preventing the knee from going deeper into its varus or valgus or, conversely, from increasing the initial angle.

The simulated biomechanical response varied across phenotypes, revealing distinct patterns of intrinsic stability that align with reported clinical distributions. Specifically, phenotypes such as CPAK III (apex distal valgus) and, to a lesser extent, CPAK VI demonstrated a marked tendency to exacerbate initial valgus alignment, while CPAK VII exacerbated baseline varus under load. Conversely, the interaction between aHKA and JLO rendered phenotypes I, II, V, and VIII substantially more resilient against secondary angular deviation. Notably, MacDessi et al. [7] reported that over 80% of asymptomatic native knees belong to groups I, II, and V, whereas these configurations are underrepresented in arthritic cohorts where less stable phenotypes (such as groups III and IV) become more prevalent. This correspondence between baseline mechanical instability and clinical prevalence suggests that native coronal phenotype may be a contributing factor in predisposing specific knee configurations to degenerative pathology.

Several methodological limitations must be noted. The knee was modeled as a simplified frontal-plane joint without explicit soft-tissue anatomy (ligaments and menisci), subjected to static axial loading rather than dynamic multi-axis kinematics. Consequently, the observed rotations represent relative baseline sensitivities across phenotypes rather than absolute in vivo magnitudes. Although these simplifications preclude direct patient-specific clinical predictions, they were necessary to enable efficient parametric screening across the entire CPAK spectrum.

## 5. Conclusions

This study presents a foundational, simplified finite element model of the lower limb designed to quickly parameterize and evaluate the Coronal Plane Alignment of the Knee (CPAK) input space. Our results indicate that specific knee phenotypes, particularly CPAK III and VII, tended to exacerbate their initial valgus or varus alignment under load. While the simplified kinematic nature of this model restricts deep clinical interpretation or direct experimental validation at this stage, it successfully isolates coronal plane mechanics and provides valuable biomechanical insights into baseline joint instability. Most importantly, this model establishes a computational framework for future exploratory studies, which will investigate how morphological changes—such as those induced by total hip arthroplasty (THA)—interact with pre-existing knee alignments to trigger the adjacent effect.

## Data Availability

Dataset available on request from the authors.

## Abbreviations

The following abbreviations are used in this manuscript:

CPAK	Coronal Plane Alignment of the Knee
LDFA	Lateral Distal Femoral Angle
MPTA	Medial Proximal Tibial Angle
THA	Total Hip Arthroplasty
TKA	Total Knee Arthroplasty
aHKA	Arithmetic Hip–Knee–Ankle Angle
JLO	Joint Line Obliquity
